# Systemic ozone therapy improves the quality of life in patients with bladder pain syndrome

**DOI:** 10.14440/bladder.2025.0002

**Published:** 2025-08-01

**Authors:** Mauro Martinelli, Aldo Glielmo, Maria Rita Licci, Daniele Romanello

**Affiliations:** 1 Department of Internal Medicine, San Pietro Fatebenefratelli Hospital, Rome, Lazio 00189, Italy; 2 Independent Researcher, Rome, Lazio 00100, Italy; 3 Department of Internal Medicine, Faculty of Medicine and Surgery, Campus Bio-Medico University, Rome, Lazio 00128, Italy

**Keywords:** Bladder pain syndrome, Ozone therapy, Redox, Systemic ozone therapy, Interstitial cystitis, Chronic pain

## Abstract

**Background:**

Bladder pain syndrome (BPS), also known as interstitial cystitis, is a chronic condition characterized by pelvic pain and urinary symptoms that severely impair patients’ quality of life (QoL). The current therapeutic options often yield suboptimal results, prompting interest in complementary treatments. Systemic ozone therapy, known for its anti-inflammatory, immunomodulatory, and analgesic effects, may represent a promising adjunctive treatment for BPS.

**Objective:**

This study aimed to evaluate the efficacy and safety of systemic ozone therapy in improving the self-perceived QoL in patients with BPS.

**Methods:**

The retrospective observational study included 40 patients diagnosed with BPS according to ESSIC criteria. All patients underwent systemic ozone therapy administered through hemotransfusion, following a standardized protocol. Outcomes were assessed using the Short Form (SF)-36 questionnaire for QoL and the Global Response Assessment (GRA) for subjective treatment efficacy. Statistical analyses evaluated changes in SF-36 domain scores and their correlation with GRA results.

**Results:**

Patients demonstrated significant improvement across all domains of the SF-36 questionnaire, with the most notable gains observed in physical functioning and mental health. The mean GRA score confirmed patient-perceived effectiveness, showing a strong positive correlation with SF-36 improvements (p<0.05). No adverse events or complications were found during the study.

**Conclusion:**

Systemic ozone therapy appears to be a safe and effective complementary treatment for BPS, significantly enhancing patients’ QoL across multiple domains. While these findings are promising, randomized controlled trials are needed to validate the results and further explore underlying mechanisms, such as the role of ozone in modulating inflammatory and neural pathways.

## 1. Introduction

Interstitial cystitis (IC) represents a chronic condition characterized by pelvic pain and urinary symptoms in the absence of other identifiable causes, such as acute or recurrent infections, nephrolithiasis, or tumors, and it affects both men and women.^1^,^2^ Most studies suggest that it is more common in females than in males, with a female predominance of 10:1 and without significant differences across ethnicities.^2^

In recent years, the International Continence Society has defined the condition as “a painful bladder syndrome” or “bladder pain syndrome” (BPS), describing it as “pelvic pain related to bladder filling, accompanied by other symptoms such as increased daytime and night-time frequency, in the absence of proven urinary infection or other obvious pathologies.” On the other hand, the term IC is reserved for BPS with histological and cystoscopic findings.^3^

The most widely utilized definitions are those of the European Society for the Study of IC/BPS (ESSIC) and that of the American Urological Association (AUA). According to ESSIC, IC/BPS is characterized by “chronic pelvic pain (>6 months), pressure or discomfort perceived to be related to the urinary bladder, accompanied by at least one other urinary symptom like persistent urge to void or frequency.” Cystoscopy is recommended to exclude other conditions. The AUA, however, defines IC/BPS as “an unpleasant sensation (pain, pressure, and discomfort) perceived to be related to the urinary bladder, associated with lower urinary tract symptoms of more than 6 weeks’ duration, in the absence of infection or other identifiable causes.”^1^-^3^

The etiology of this condition remains unknown but is likely multifactorial.^4^ A bladder insult caused by increased urothelial permeability, immunological and neurogenic abnormalities, or pelvic floor dysfunction may result in disruption of the apical cell layer of the urothelium, which protects the bladder from infection in healthy individuals.^3^ This damage triggers inflammation, leading to a self-perpetuating process and resulting in chronic bladder pain. Consequently, other chronic pain conditions, such as irritable bowel syndrome or fibromyalgia, may precede or follow BPS.^4^

Pathological findings on cystoscopy with hydrodistension include Hunner’s lesion and glomerulations. Hunner’s lesion, considered distinctive of IC, is present only in a small portion of IC/BPS patients (4 – 10%). Based on their presence, IC/BPS is classified into ulcerative and non-ulcerative forms.^5^

Hunner’s lesions are characterized by deep damage to the mucosa and submucosa caused by bladder distension. They present as red mucosal areas with small vessels radiating toward a central scar with fibrin deposit.^3^

Moreover, IC/BPS significantly impacts daily activities, physical health, and the overall quality of life (QoL) of affected individuals.^6^

The treatment is primarily symptomatic and includes several approaches, depending on disease severity and patient response.^7^ Non-invasive options involve lifestyle modifications and stress management, as stress can exacerbate symptoms.^8^ Oral medications such as tricyclic antidepressants, antihistamines, and pentosan polysulfate sodium are commonly used to alleviate pain and urinary symptoms. Intravesical therapies, including the instillation of agents such as dimethyl sulfoxide, hyaluronic acid, or heparin into the bladder, aim to restore the damaged urothelial barrier.^9^ In severe or refractory cases, more invasive treatments, such as cystoscopy with hydrodistension or surgical procedures like cystectomy with urinary diversion, may be considered.^10^ Due to the heterogeneity of the condition, the therapeutic approach must be individualized, often combining multiple strategies to improve the patient’s QoL.

Given the limitations of current treatments, alternative therapies such as ozone therapy have been explored for their anti-inflammatory and analgesic effects. Ozone therapy is a complementary treatment extensively used in pain management.^11^ Due to its safety profile and lack of pharmacological interactions, ozone therapy can be administered alongside other conventional treatments, often acting as an enhancer.^12^ Beyond its well-documented analgesic effects, achieved through both topical and systemic administration, emerging evidence suggests that ozone therapy may also have antidepressant properties, opening new avenues for research into its broader therapeutic potential.^13^ Several studies have demonstrated the anti-inflammatory effects of ozone therapy, which are particularly relevant in IC/BPS, where chronic inflammation contributes to urothelial damage, neurogenic hypersensitivity, and self-perpetuating pain cycles. Moreover, there is evidence that ozone therapy may positively influence patients’ perceived QoL.^3^

The aim of this study was to evaluate the effect of ozone therapy on the self-perceived QoL in patients affected by BPS.

## 2. Materials and methods

This study was conducted at the Ozone Therapy Center of San Pietro Fatebenefratelli Hospital in Rome, Italy. The ethics committee acknowledged the data analysis for the purposes of this retrospective study. A total of 40 female patients, aged between 28 and 81 years, were included in the analysis. Among them, 31 had their condition confirmed through biopsy, and 23 presented with comorbidities. Data were collected from patients who had been treated or diagnosed between January 01, 2019, and December 31, 2023. All patients had received a clinical diagnosis of BPS at least 6 months before the initiation of ozone therapy, confirmed by cystoscopy and uroflowmetry. Inclusion and exclusion criteria are detailed in Table 1. All patients had previously undergone standard treatment without significant benefits.

**Table 1:** Inclusion and exclusion criteria

Inclusion criteria	Exclusion criteria
Clinical diagnosis of IC/BPS	Severe deficit of G6PDH
Diagnosis confirmed by cystoscopy and uroflowmetry	Severe anemia
No benefit from standard treatment	

Abbreviations: BPS: Bladder pain syndrome; G6PDH: Glucose-6-phosphate dehydrogenase; IC: Interstitial cystitis.

For all patients included in this analysis, normal glucose-6-phosphate dehydrogenase levels had been confirmed before receiving ozone therapy, in accordance with standard clinical protocols. This assessment was performed to ensure patient safety and avoid complications related to oxidative stress. In addition, informed consent for clinical procedures, including ozone therapy, was obtained at the time of treatment, in line with institutional and ethical guidelines. Ozone therapy was prepared according to the “Nuova FIO” guidelines and standard clinical practices, ensuring consistency with and adherence to quality standards. The procedure involved the extraction of a defined volume of venous blood, collected into a sterile, citrate-treated container. A calibrated ozone-oxygen mixture was then introduced into the container under controlled conditions to achieve the desired concentration, ensuring optimal gas–liquid homogeneity. The blood was gently agitated to facilitate the absorption of ozone, allowing for adequate interaction with erythrocytes and plasma components. After the ozone enrichment phase, the treated blood was promptly reinfused into the patient through the same venous access, using aseptic techniques to prevent contamination. Vital signs were monitored throughout the procedure to detect any adverse reactions and ensure patient stability.^14^

As part of this retrospective analysis, data from the Short-Form 36 (SF-36) questionnaire were reviewed. The SF-36, a validated tool for assessing QoL, consists of 36 items across eight domains, providing a comprehensive measure of patients’ overall health status. Originally administered during routine clinical care, the questionnaire served a dual purpose: it provided physicians with insights into the therapy’s effectiveness and allowed patients to track and reflect on their progress over time. This dual utility enhances the relevance of SF-36 data, particularly in the absence of reliable biological markers for conditions like IC/BPS, where patient-reported outcomes are essential for evaluating therapeutic impact.

The eight SF-36 domains are:

- Physical functioning (10 questions)
- Social functioning (4 questions)
- Bodily pain (2 questions)
- Role limitations due to emotional problems (3 questions)
- Role limitations due to physical problems (4 questions)
- General health perceptions (1 question)
- Mental health (5 questions)
- Vitality (4 questions).

The SF-36 yields a score ranging from 0 to 100, with higher scores indicating a better QoL. A Student’s *t*-test was used to analyze variations in these scores before and after therapy and to compare results between different groups of patients.

The ozone therapy protocol consisted of 14 sessions over 3 months. The treatment schedule was as follows: two sessions per week for the first 2 weeks, followed by one session per week for the remaining 10 weeks. Blood was collected and mixed in appropriate bags with an oxygen–ozone (O_2_–O_3_) mixture, then gently shaken for 2 min. Specific details of the therapeutic protocol are presented in Table 2.

**Table 2:** Therapy protocol

Time	Blood	Ozone volume (concentration 40 mcg/mL)	Total dose
1^st^ session	180 mL	180 mL	7200 mcg
2^nd^ session	200 mL	200 mL	8000 mcg
3^rd^ session	220 mL	220 mL	8800 mcg
4^th^ – 15^th^ session	240 mL	240 mL	9600 mcg

As part of this retrospective analysis, data from the Global Response Assessment (GRA) were reviewed to evaluate the overall effectiveness of the treatment as perceived by patients. The GRA, a comprehensive tool to assess patients’ subjective perception of therapeutic effectiveness, had been administered at the conclusion of therapy as part of routine clinical care. These data provided a direct measure of patients’ perceived improvement, complementing the objective results from the SF-36 questionnaire and enabling a thorough assessment of therapeutic impact.

To further explore the consistency between subjective and objective outcomes, a correlation analysis was performed between GRA scores and SF-36 results using a Pearson correlation test.

## 3. Results

Based on the reviewed clinical records, no patients were excluded from the analysis due to adverse effects, suggesting that the treatment was well-tolerated across the cohort.

The SF-36 showed a mean score of 36.76 (standard deviation [SD] 19.38) before therapy and a mean score of 55.47 (SD 17.66) after therapy, with a mean improvement of 18.71 (SD 14.3).

**Figure 1:**
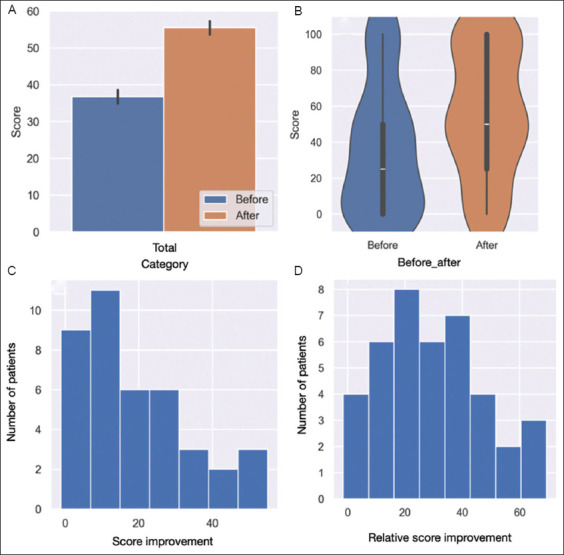
SF36 scores before/after ozone therapy. (A) Box plot showing the distribution of Short-Form 36 (SF-36) scores before and after ozone therapy. (B) Violin plot illustrating the distribution of SF-36 scores before and after ozone therapy. The plot shows the density of the data at different score levels, with a central white line denoting the median and a thick black bar representing the interquartile range. (C) Histogram showing the distribution of SF-36 score improvements among patients after ozone therapy. The x-axis represents the degree of improvement in scores, while the y-axis indicates the number of patients in each category. (D) Histogram depicting the distribution of relative SF-36 score improvements among patients after ozone therapy. The x-axis represents the percentage of relative score improvement, while the y-axis indicates the number of patients in each category.

A significant increase (*p*<0.001) in the overall median SF-36 scores was observed (Figure 1A). A violin plot with a kernel density estimator was also generated, showing three different percentiles (Figure 1B). The distribution of SF-36 score and the distribution of relative SF-36 score improvements are shown in Figure 1C and Figure 1D

**Figure 2:**
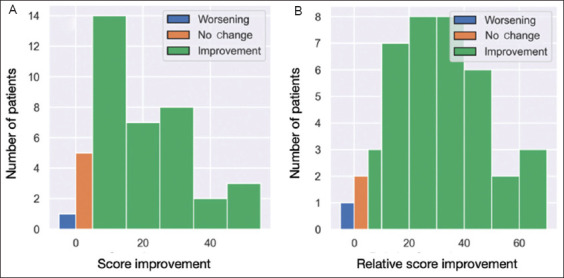
Score improvement. (A) Histogram categorizing patients based on Short-Form 36 (SF-36) score improvement after ozone therapy, using a 5-point relative improvement cutoff. Patients are classified into three groups: improvement (green), no change (orange), and worsening (blue). The y-axis represents the number of patients in each category. (B) Histogram categorizing patients based on relative SF-36 score improvement after ozone therapy, using a 5% relative improvement cutoff. Patients were classified into three groups: improvement (green), no change (orange), and worsening (blue). The x-axis represents the percentage of relative score improvement, while the y-axis indicates the number of patients in each category.

We arbitrarily defined a threshold of 5 points on the SF-36 scale to signify a meaningful variation. Based on this criterion, 34 patients (85%) demonstrated improvement, five patients (12.5%) showed no change, and one patient (2.5%) experienced a worsening of their condition (Figure 2A). This result is confirmed by the relative score improvement (Figure 2B).

**Figure 3:**
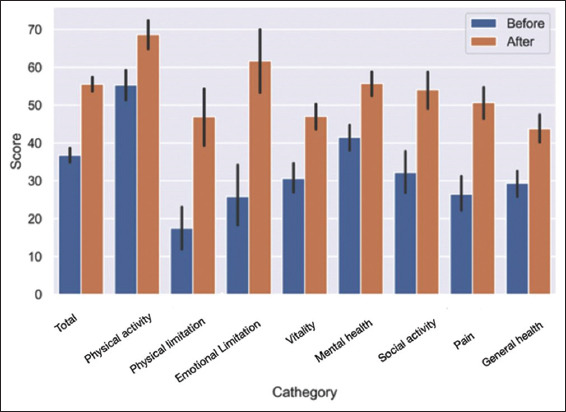
Bar chart comparing Short-Form 36 domain scores before and after ozone therapy. Each category represents a different health domain, with scores measured before (blue) and after (orange) treatment. Error bars are indicative of standard deviations.

When analyzing each of the eight SF-36 domains individually, a statistically significant improvement was noted in all areas (*p*<0.001) (Figure 3).

**Figure 4:**
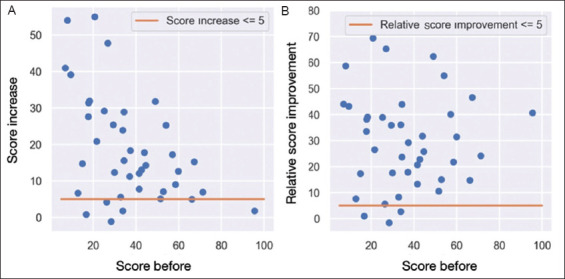
Scatter plot pre and post-treatment. (A) Scatter plot showing the relationship between pre-treatment Short-Form 36 (SF-36) scores and absolute score increases after ozone therapy. Each dot represents an individual patient, with the x-axis indicating the initial SF-36 score and the y-axis showing the absolute improvement. The horizontal line represents a 5-point increase threshold. (B) Scatter plot showing the relationship between pre-treatment SF-36 scores and relative score improvements after ozone therapy. Each dot represents an individual patient, with the x-axis indicating the initial SF-36 score and the y-axis showing the relative improvement. The horizontal line represents a 5% relative improvement threshold.

The most substantial improvements in absolute terms were observed among patients with lower baseline scores at T0 (Figure 4A), a finding that was confirmed by the relative score improvements (Figure 4B).

**Figure 5:**
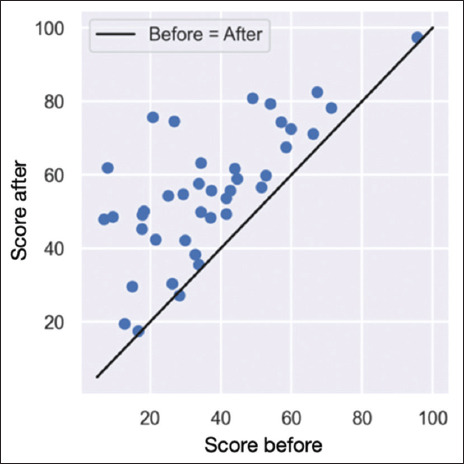
Scatter plot comparing pre-treatment and post-treatment Short-Form 36 (SF-36) scores after ozone therapy. Each dot represents an individual patient. The diagonal line represents the “Before = After” reference, where points above the line indicate an improvement in SF-36 scores.

This scatter plot is included to provide a clearer visualization of the results, illustrating individual changes in SF-36 scores before and after therapy (Figure 5).

The GRA further corroborated the positive outcomes of the therapy. Specifically, 30 patients showed moderate to significant improvement, five patients exhibited mild improvement, and five patients had no improvement. Importantly, no patients suffered from a deterioration of their condition.

**Figure 6:**
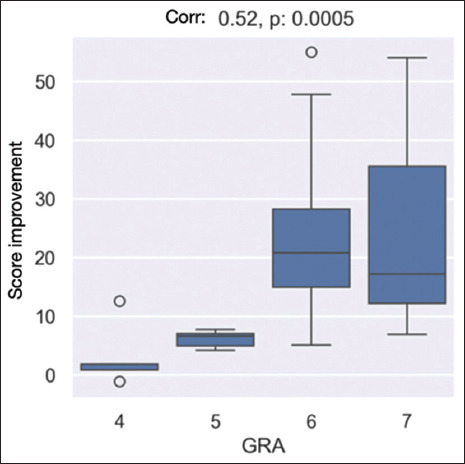
Box plot illustrating the correlation between Global Response Assessment (GRA) scores and Short-Form 36 score improvement after ozone therapy. Higher GRA scores correspond to greater perceived improvement. The correlation coefficient (r = 0.52, p = 0.0005) indicates a statistically significant positive association.

The correlation between the results of the GRA and the SF-36 scores was found to be statistically significant (*p*<0.001), reinforcing the validity of the observed improvements in patient outcomes (Figure 6).

## 4. Discussion

In this study, we observed a statistically significant improvement in the self-perceived QoL of 40 patients affected by BPS, as measured by the SF-36 questionnaire. The GRA, used to evaluate patients’ subjective perception of treatment effectiveness, verified these results. In addition, the correlation between the GRA scores and the SF-36 outcomes was also statistically significant.

No adverse effects or reactions were reported during the treatment, reinforcing the safety profile of ozone therapy.^12^,^15^ However, we acknowledge the limitations of this study, including its retrospective design, the lack of a placebo or control group, and the relatively small sample size. These factors may introduce potential biases, particularly in patient selection and the interpretation of subjective outcomes. Moreover, the reliance on patient-reported outcomes, such as the SF-36 and GRA, introduces the possibility of subjective bias, although the strong correlation between these tools suggests internal consistency. Finally, as the study did not include long-term follow-up, the durability of the observed improvements remains unknown. Despite these limitations, our findings align with previous evidence supporting the effects of ozone therapy on self-perceived QoL.^9^ The significant improvements observed across all domains of the SF-36 suggest that ozone therapy may exert both psychological and physical effects. This may be attributed to its known anti-inflammatory properties, its impact on micro-vascularization, and its ability to enhance tissue oxygenation. In addition, the observed improvement in mental health could reflect the hypothesized antidepressant effects of ozone, a mechanism increasingly supported in the literature. For instance, we developed a theoretical model proposing the involvement of metabotropic glutamate receptors in mediating these antidepressant effects, providing a potential molecular explanation for the outcomes observed.^16^ While these mechanisms remain speculative in the context of our study, they warrant further investigation.

Interestingly, the treatment seemed to exert a stronger impact on patients with lower baseline scores, possibly because standard therapies yielded less benefits in these individuals. As a complementary treatment, ozone therapy may enhance outcomes, particularly in cases where conventional treatments have shown limited effectiveness. We chose a 5% cutoff for the variation in SF-36 scores, as it provides a sufficiently sensitive threshold to detect gradual improvements in QoL while allowing for a detailed categorization of patients. This stratification enables a more precise evaluation of post-therapy improvements, facilitating both clinical and statistical interpretation of the data, especially in the absence of a universally recognized minimal clinically important difference for SF-36 in patients with BPS.

Based on our findings and considering its favorable safety profile, ozone therapy could be considered a complementary treatment option for BPS in specific cases. The statistically significant improvements observed across all domains of the SF-36 suggest potential benefits that need further exploration. However, additional studies, particularly prospective randomized controlled trials, are necessary to confirm these findings and to better define the role of ozone therapy in the management of BPS.

## 5. Conclusion

Ozone therapy appears to be a safe and promising complementary treatment for BPS, with the potential to improve QoL. However, its efficacy requires further validation through randomized controlled trials, given the study’s retrospective design, small sample size, and lack of a control group.

## Data Availability

Data obtained from this study are available from the corresponding author upon reasonable request.
